# Influence of proximal femoral morphology on osteointegration of the AMIS uncemented femoral stem in modern hip arthroplasty

**DOI:** 10.1186/s42836-024-00274-y

**Published:** 2024-10-11

**Authors:** Maxime Maton, Emile Vandromme, Tatiana Charles, Bilal Kapanci, Marc Jayankura

**Affiliations:** Orthopedics and Traumatology Department, Erasme Hospital, Brussels, 1070 Belgium

**Keywords:** Hip arthroplasty, Femoral morphology, Osteointegration

## Abstract

**Background:**

Indications for total hip arthroplasty (THA) have evolved over recent decades, with a growing trend toward operating on younger and more active patients. With this shift in patient demographics, there has been a change in femoral stem designs and proximal femoral morphology encountered. This study aimed to evaluate the potential correlation between femoral stem osteointegration and proximal femoral morphology using the Dorr and Noble classifications.

**Materials and methods:**

We conducted a retrospective review of 122 uncemented femoral stems (AMIstem Medacta, triple tapered). The demographic data analyzed included sex, age, stem size, and surgical indications. Preoperative radiographs were reviewed to determine the Dorr classification as well as the canal-to-calcar isthmus ratio, cortical index, and canal flare index. Postoperative radiographs were carefully reviewed to identify the presence of potential postoperative radiolucencies. Inter- and intra-observer agreements for these parameters were also analyzed.

**Results:**

Significant radiolucencies (> 2 mm) were found in 19.5% of the patients. Of all the variables analyzed, the cortical index was the only parameter significantly associated with the appearance of clinically significant radiolucency, with a threshold value of 0.62.

**Conclusions:**

The appearance of radiolucencies is multi-factorial. Nevertheless, in this study, a high cortical index (> 0.62), representing the champagne flute morphology, was found to be associated with the development of significant radiolucencies.

## Introduction

Attempts at surgical treatment of osteoarthritis date back to the late 19th century [1, 2]. This was revolutionized in the 1960s by Prof. John Charnley, who was considered the father of modern hip arthroplasty and introduced the concept of low-friction arthroplasty [3]. Hip arthroplasty has evolved, in all its aspects, over recent decades, particularly in the fixation of the femoral stem, with a current trend toward the use of non-cemented implants [4].

Several authors have evaluated femoral morphology to optimize surgical indications, postoperative results, implant fixation, and osteointegration in total hip replacements. However, it wasn’t until 1988 that Noble et al. [5] exhibited a particular interest in the morphology of the proximal femur. They introduced the canal flare index, categorizing proximal femurs into three different morphological groups: stovepipe, normal, and champagne flute proximal femurs.

Following this, Dorr et al. [6] attempted to correlate the morphological characteristics of the proximal femur observed through X-ray analysis with femoral bone structure and cellular activities. Femurs were classified into three types: Type A, prevalent in young, heavy men, characterized by thick cortices and lower porosity; Type B, characterized by thinner medial and posterior cortices and higher porosity than Type A; and Type C, more frequently encountered in older women, characterized by very thin cortices. The study revealed that patients with Type C femurs who underwent hip arthroplasty experienced increased thigh pain, adaptive bone remodeling, and reduced prosthetic support density. Nevertheless, the clinical outcomes were comparable to those with Type A and B femurs during a two-year follow-up. This work dates back to 1993, a period associated with a significant prevalence of Dorr Type C femurs among patients undergoing hip arthroplasty.

Recently, demographic shifts have been observed, as patients who benefit from total hip arthroplasty (THA) tend to be younger and more active [1, 2]. This trend might be associated with differences in femoral morphology encountered during surgeries, potentially leading to an increased prevalence of Type A or champagne-flute femurs.

Despite the excellent results of contemporary prosthetic designs, failures are still being described in the literature. Some authors have pointed out a possible association between femurs displaying a Dorr Type A morphology and a greater incidence of osteointegration failures [7–9].

In this context, it might be interesting to re-investigate the association between femoral morphology and osteointegration of modern stem designs. The primary objective of this study was to assess the radiological integration of modern stem designs and to explore the potential impact of proximal femoral morphology on the occurrence of postoperative radiolucencies following total hip arthroplasty (THA).

## Materials and methods

This retrospective study was conducted from October 2010 to October 2017.

We investigated the initial 200 patients (involving 209 hips) who underwent total hip arthroplasty (THA) with an AMIStem manufactured by Medacta® between October 2010 and June 2013. The surgical procedures, examined in our study, were conducted by the senior author (MJ), utilizing an anterior approach with leg support.

As mentioned above, we included patients who underwent elective primary THA with an AMIS (Medacta®) uncemented stem.

The exclusion criteria were as follows: (1) the use of a stem other than the one previously described, whether cemented or uncemented; (2) THA indicated for femoral neck fractures or sequelae of pediatric hip pathologies; and (3) a history of postoperative dislocation, periprosthetic fracture, infection, or a follow-up period less than twelve months.

The stem implanted was an AMIStem uncemented, collarless stem manufactured by Medacta® (Strada Regina, 6874 Castel San Pietro, Switzerland). This stem features a triple-tapered design and reduced lateral flare. It is made from a titanium and niobium alloy with an 80-µm hydroxyapatite coating. The bearing surfaces used were ceramic-on-ceramic bearings.

### Radiological analysis

All radiographs were analyzed by two independent physicians (MM and BK) to quantify intra- and inter-observer agreement. All patients benefited preoperatively from the frontal hip and pelvic radiographs calibrated by a ruler or a metallic head with a diameter of 28 mm, allowing for preoperative planning. Radiographs were used for the characterization of femoral morphology with the following measures.

### Preoperative measurements of femoral morphology

The canal flare index (CFI) corresponds to the ratio of the intramedullary diameter at 20 mm proximal and 100 mm distal to a reference line drawn in the middle of the lesser trochanter [4].

The canal-to-calar isthmus ratio (CC) represents the dimensional ratio of the intramedullary canal isthmus to the calcar isthmus. The intramedullary canal isthmus was measured 100 mm from the reference line. The calcar isthmus was measured after constructing 2 straight lines (medial and lateral) passed through 2 points located on the endosteal margin 30 mm and 100 mm from the reference line. The distance between the intersections of these two lines and the reference line corresponds to the distance of the calcar isthmus.

**Fig. 1:**
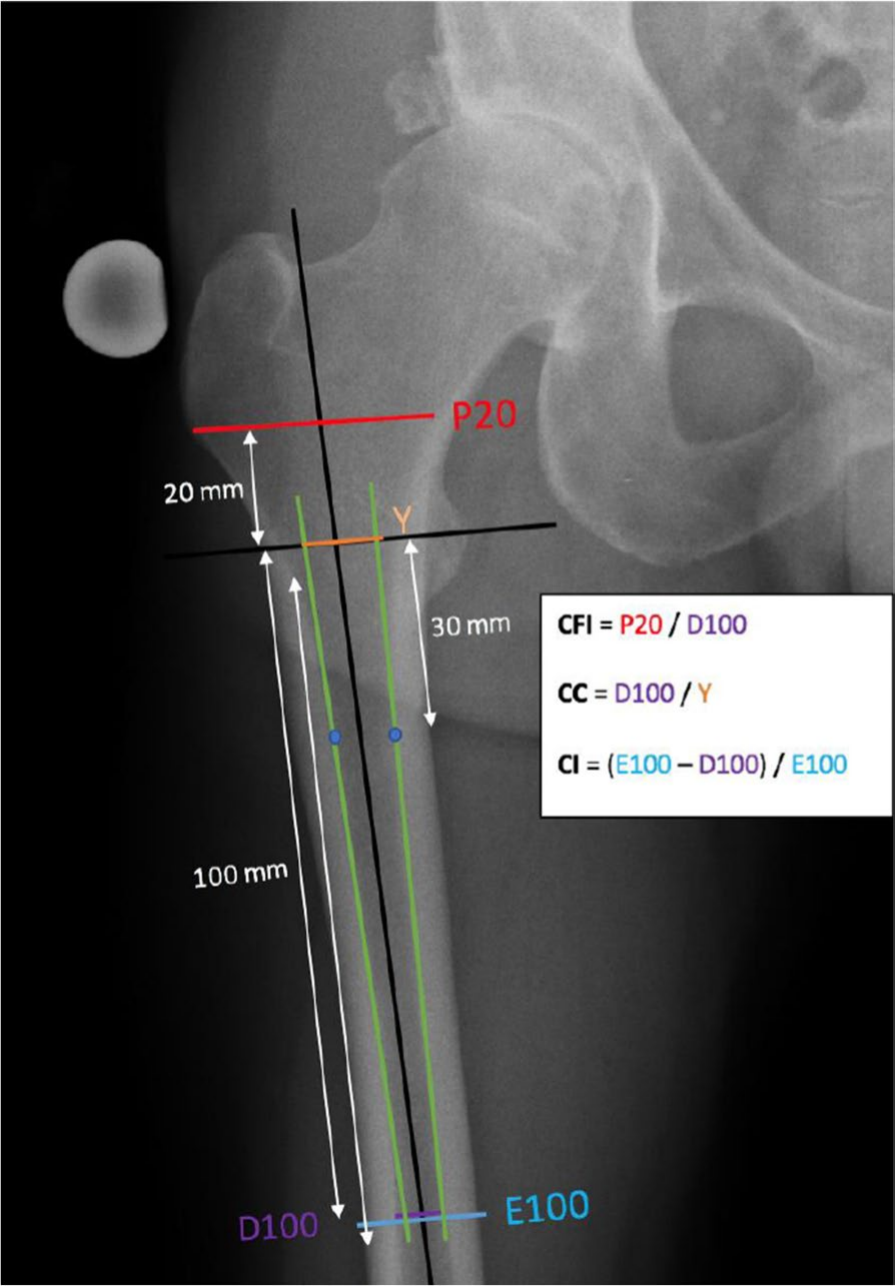
Representation of the measures used on the preoperative radiographs. Preoperative radiograph of a right hip, frontal view CFI = P20/D100; CC = D100/Y; CI = (E100 – D100)/E100; CFI = canal flare index; CC = canal to calcar isthmus; CI = cortical index

The cortical index (CI) is the ratio between the diaphyseal diameter (FD), from which we subtract the intramedullary diameter (MD), and the diaphyseal diameter (CI = FD-MD/FD) (Fig. 1). Both diameters were measured 100 mm distal to the reference line. The femurs were classified according to Dorr’s classification by using the last two measures [6].

### Postoperative measurements

All postoperative radiographs were reviewed for signs of osteointegration defects around the femoral stem. The presence of any radiolucency around the femoral implant was characterized according to Gruen’s zone [12]. The threshold for radiologically significant radiolucency was a width of 2 mm. For this analysis, we calibrated the radiographs before measurements using two methods: a graduated ruler positioned alongside the patient’s thigh and a 28-mm ball, which served as a substitute for the ruler.

### Statistical analysis

Comparisons between patients with and without any radiologically significant radiolucency were performed using descriptive statistics. The associations between the occurrence of radiolucency and demographic or radiological parameters were tested using chi-square tests and Mann–Whitney tests for independent samples. Logistic regression using ROC curves was employed when a parameter was found to be significantly associated with the appearance of a radiolucent border, followed by a Youden index to determine if a threshold value might be found. Statistical significance was defined by a *P*-value ≤ 0.05.

### Power analysis

Since this study was of a retrospective nature, we had not conducted a power analysis. Nevertheless, we calculated our power in a post-hoc analysis, using our results. Hypothesizing a prevalence of radiolucency of 20% among patients with a cortical index lower than 0.62 and of 40% among patients with a CI higher than 0.62, we calculated that, with a type I error of 5% and a power of 80% (type II error), 150 patients would be needed in the study.

## Results

Of the 209 total hip arthroplasties, 86 were excluded from the analysis for the following reasons: 5 had cemented stems, 6 had a history of infection, 2 were performed for femoral neck fractures, 1 had an episode of dislocation, 7 had peri-prosthetic fractures (intra- or postoperative), 5 had proximal femoral deformities (post-traumatic or sequelae of pediatric diseases), and 57 received a follow-up lasting for less than 12 months at the time of the study or lost to follow-up. Upon application of the inclusion and exclusion criteria, a total of 122 hip replacements performed in 115 patients remained for analysis. There were 66 males (54%) and 56 females (46%). The mean age was 59 years (± 14.2 SD). The indications for THA were primary osteoarthritis in 96 patients (78%) and avascular necrosis of the femoral head in 26 patients (22%). The mean follow-up time lasted for 36 months [12–64 months]. The male/female ratio in this series was virtually one to one: 66 males vs. 56 females. Sex was not significantly associated with the appearance of any radiolucency.

Significant radiolucency (> 2 mm) was found in 24 hips (19.5%). The surgical indications for the implantation of these prostheses were primary osteoarthritis in 21 patients (87.5%) and avascular necrosis in 3 patients (12.5%), with no significant difference between patients with and without radiolucency. These patients were compared to their counterparts who did not show any significant radiolucency on postoperative X-rays in terms of demographics (age, BMI, sex) (Table 1) and morphological parameters of the proximal femur (CFI, CC, and CI) and stem size (Table 2). The only variable showing a statistically significant difference between the two groups was the cortical index (CI). A greater CI was significantly associated with the presence of significant postoperative radiolucencies (*P*-value: 0.03).

**Table 1:** Demographic data

	With radiolucency (*n* = 24*)*	Without radiolucency (*n* = 99*)*	*P*-value
Patient age^a^	59.50 (50.49–67.76)	60.00 (58.00–63.00)	0.39
BMI^a^	25.82 (23.28–27.44)	26.31 (24.54–27.71)	0.52

^a^The values are given as median (95 percentile range)

**Table 2:** Morphological data

	With radiolucency (*n* = 24*)*	Without radiolucency (*n* = 99)	*P*-value
Canal Flare Index (CFI)^a^	3.78 (3.41–4.14)	3.69 (3.51–3.77)	0.30
Canal to Calcar isthmus ratio (CC)^a^	0.61 (0.59–0.69)	0.63 (0.60–0.63)	0.93
Cortical Index (CI)^a^	0.63 (0.58–0.67)	0.59 (0.58–0.61)	**0.03**
Stem size^a^	4 (3.00–4.65)	3 (3.00–4.00)	0.78

^a^The values are presented as median (95 percentile range)

In this context, we conducted a ROC curve analysis to examine the effect of the cortical index on the risk of developing radiolucencies.

**Fig. 2:**
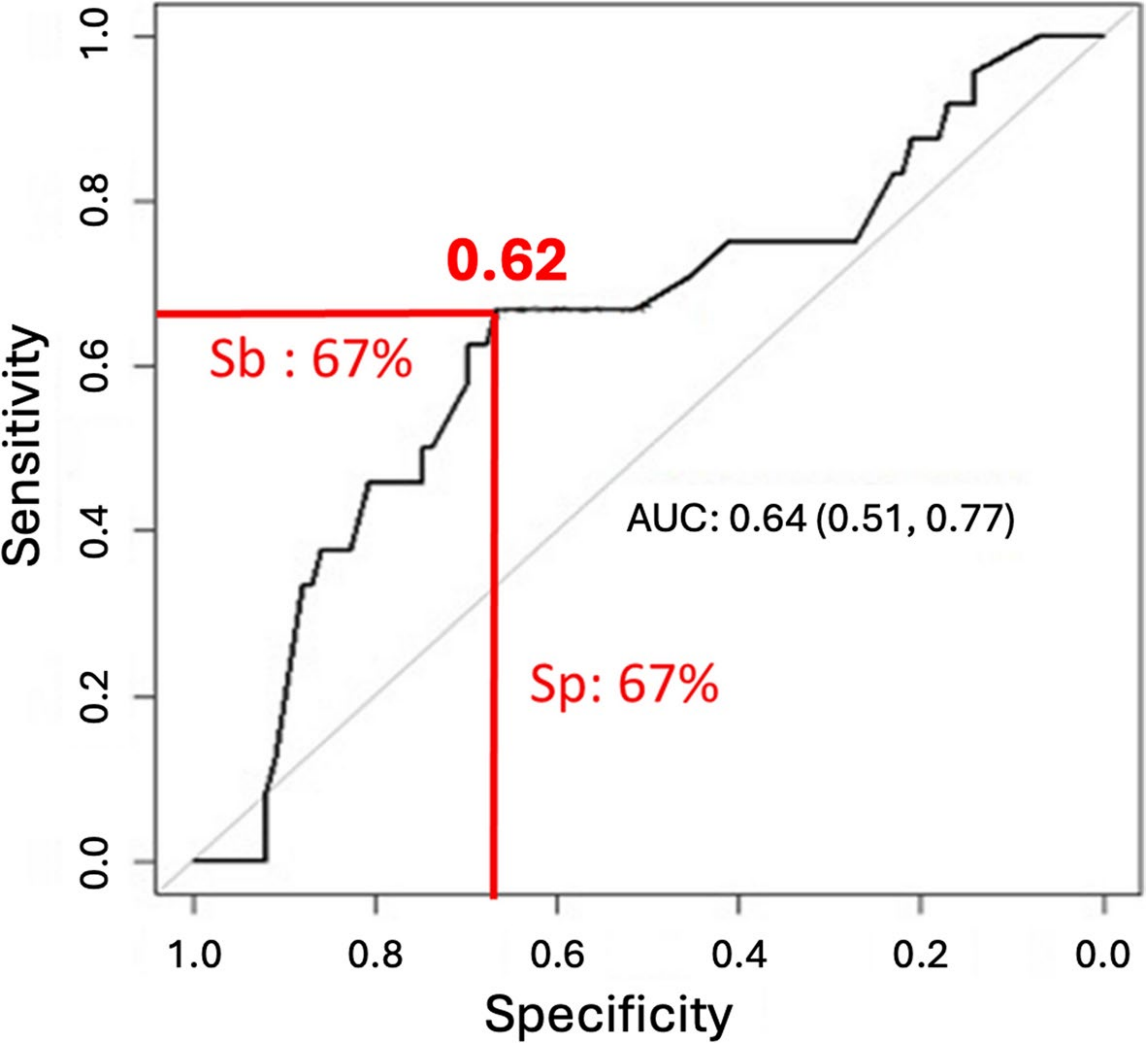
Sensitivity and specificity of the CI. The Youden index determined a threshold value of 0.62 for the CI with a sensitivity of 67% and a specificity of 67%

A cortical index with a threshold value of 0.62 was found to be associated with the presence of postoperative radiolucency, with a sensitivity of 67% and a specificity of 67% (Fig. 2).

**Fig. 3:**
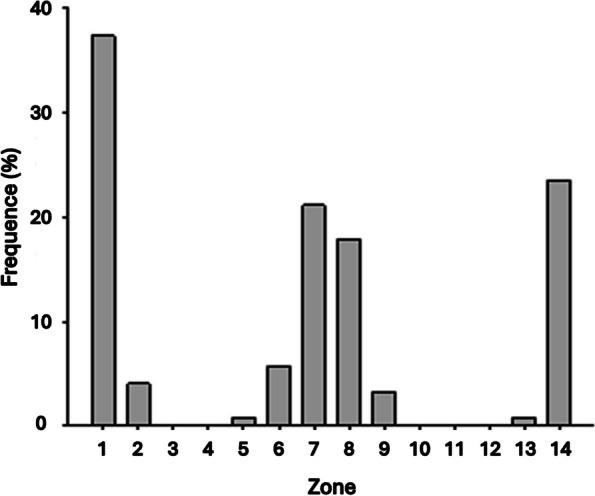
Analysis of radiolucencies according to Gruen’s zones. Distribution of radiolucencies according to the Gruen zones, demonstrating a predominance in zones 1, 7, and 8 and no predominance in zones 3, 4, 10, 11 and 12

Radiolucencies were predominantly described in proximal femoral zones 1, 7, 8, and 14 according to Gruen, and less frequently (< 10%) in zones 2, 5, 6, 9, and 13. No radiolucent zones were found in the other zones (3, 4, 10, 11, and 12) (Fig. 3).

**Fig. 4:**
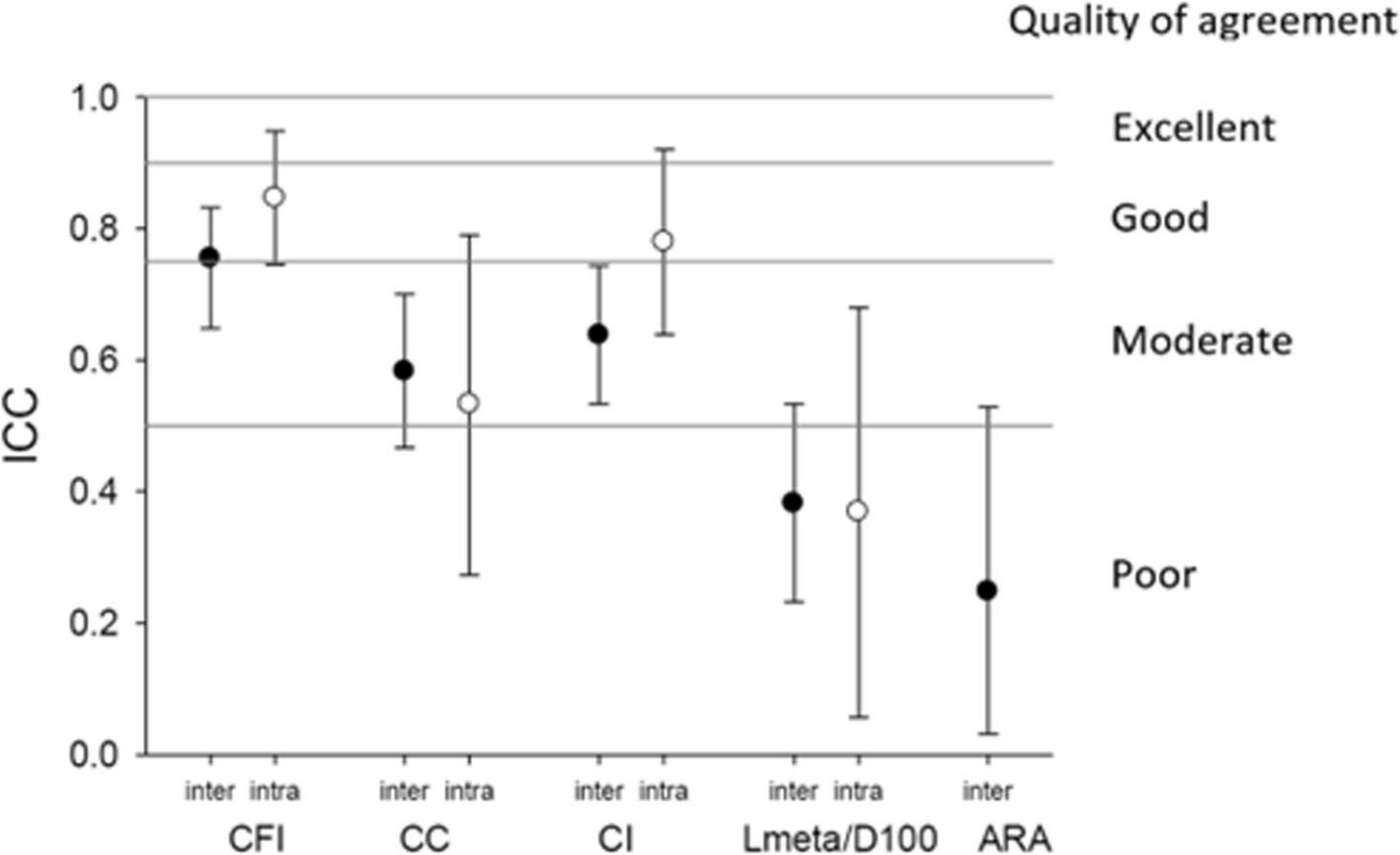
Diagram highlighting inter- and intra-observer agreement

Inter- and intra-observer variability was also analyzed for the morphological measures (CFI, CC, CI) and the appearance of radiolucencies. Intraclass correlation (ICC) showed an acceptable or good inter-observer correlation for CFI, CC, and CI, regardless of their experience (Fig. 4). The analyses of postoperative radiolucency showed good reproducibility, with a Cohen’s kappa index of 0.60 for any radiolucency and 0.67 for any significant radiolucency.

## Discussion

The presence of radiolucencies on early postoperative radiographs after total hip arthroplasty (THA), in combination with clinical symptoms, may indicate inadequate osteointegration of the femoral stem [10].

In this particular series, we observed significant radiological radiolucencies in 19.5% of patients, which aligned with findings in the literature, with prevalence rates ranging from 0 to 17% [11]. To the best of our knowledge, few other studies have investigated the association between proximal femoral morphology and modern hip stem designs.

Femoral morphology and its potential impact on hip replacement surgery, as well as histological characteristics, were initially described in the 1990s [6].

More recently, Cooper et al. [7] emphasized the significance of femoral morphology in the context of femoral stem osteointegration. Their findings indicated that femurs with a smaller canal flare index (stovepipe femurs), male sex, and the use of larger stem sizes increasing femoral filling in the middle and distal thirds of the femur were potentially associated with failures in femoral stem osteointegration during the early postoperative period. The authors suggested that the stem design (Stryker TMZF) could account for the lack of osteointegration in stovepipe femurs. It was specifically noted that the distal portion of the stem exhibited a relative increase in width compared to the proximal portion, possibly leading to distal rather than proximal wedging and fixation when using larger stems in larger femoral canals (Dorr C femur).

In contrast, Ishii et al., in 2016 [9], reviewed 81 hips of Asian females using the same stem design, and reported a significant association between a greater canal flare index (champagne flute femur) and osteointegration failure based on radiological findings. Additionally, they described a smaller canal fill ratio in femurs showing signs of osteointegration (spot welds). This measure evaluated the disparity in canal filling between the proximal and distal segments of the femur. A smaller canal fill ratio was defined by improved filling in both the proximal and distal regions of the femur and was associated with better osteointegration.

The contradictory results in these two studies, despite the use of the same stem, require careful examination. The first study (Cooper et al. [7]) demonstrated an increased incidence of osteointegration failure in Dorr Type C femurs, while the second study reported similar findings in Dorr Type A femurs (Ischii et al. [9]).

In a study by Cooper, it is plausible that the stem design played a significant role in the observed results. As described by the author, an increase in implant size was associated with a greater increase in the distal width of the stem compared to the proximal width. The use of this implant type could, therefore, result in distal fixation of larger sizes, which are mainly used in type C femurs.

In contrast, in the research conducted by Ishii et al., distal loading could be directly associated with femoral morphology (Dorr A—champagne flute) and might be a contributing factor to osteointegration failure. This observation is in agreement with our findings.

A study conducted in 2019 by Park et al. [8] reviewed 1089 hips, in which a titanium, cementless Bencox stem was implanted. They investigated and compared the clinical and radiographic outcomes of THA in patients with Dorr type A femurs vs. type B femurs. Statistical analysis revealed a significant difference between the groups, with more radiolucent lines observed around the femoral stem and lower survivorship rates in patients presenting with type A femurs during a mean follow-up period of 7 years. Hips with radiolucency exhibited significantly worse clinical outcomes, as indicated by the Harris hip score. Our results also are in line with the conclusions drawn by Park et al.

These findings are extremely interesting in the current context of hip arthroplasty. The greater prevalence of Dorr Type A femurs can be explained by age-related shifts as well as increased osteoporosis prevention measures and bisphosphonate use in the elderly population.

The increased prevalence of Dorr Type A femurs within the hip replacement population has been documented by various studies [8]. For instance, Nash et al. [12] reported a prevalence rate of 16.9% for Type A femurs in their series of 172 patients, with an average age of 85 years. In comparison, after examining 871 patients, Kim et al. [13] reported a prevalence rate of 85%, with a mean age of 52.9 years. Changes in femur geometry, structure, and morphology related to age have also been the subject of computed tomography studies [14, 15].

Confronted with this paradigm shift, it seems only natural that the once-dominant question regarding the evolution of hip prostheses in Type C femurs should evolve toward an investigation of the outcomes of current prostheses in Type A femurs, facilitating a more in-depth understanding of the current challenges in the field.

In our study, we did not find an association between the canal flare index (CFI) and radiographic signs of osteointegration failure. However, the canal index (CI) was significantly associated with the occurrence of radiological signs of osteointegration failure. According to the Youden index, a CI of 0.62 or greater was linked to an increased likelihood of postoperative radiolucency occurring 5 years after surgery. This CI value corresponds to a champagne flute femur, classified as type A according to Dorr [5]. Our results are coincident with the available literature showing that this particular femoral morphology may carry a greater risk of osteointegration failure in the early postoperative period when classic tapered stems are used.

The concept of proximal–distal mismatch, as described by Ishi et al. [14], may explain this osteointegration failure. According to this theory, when implanting classic tapered stems into very narrow champagne flute femurs, the stem may undergo distal wedging and fixation without adequately filling the wider metaphysis. This configuration could lead to proximal micromovements, ultimately resulting in proximal osteointegration failure of the stem. Considering that, in our study, radiolucencies were predominantly noted in Gruen’s zones 1, 7, and 8, corresponding to the proximal regions, thus supporting this theory.

Notably, our study was the first to introduce a radiological parameter showing a threshold value significantly associated with the appearance of postoperative significant radiolucency in contemporary THA practice. The relatively low sensitivity and specificity of this value may be attributed to the limited size of our study sample and might improve with increased sample size.

The reproducibility of radiological analysis and measurements is crucial to assisting practitioners in their daily practice. The reproducibility of the Dorr classification, which relies on a qualitative assessment of the femur, has been evaluated in different studies with conflicting results. Wellman et al. [16] described the Dorr classification as reproducible, with minimal variations between observers. In contrast, Mazhar et al. reported that reproducibility depended on the observer’s level of experience [17].

Nakaya et al. [18] conducted a review of quantitative measurements for classifying femoral types as described by Dorr. They concluded that the most suitable indices for classification, using plain hip radiographs, were the CI on anteroposterior and lateral views. Utilizing these quantitative indices improved inter- and intra-examiner reproducibility, making them valuable tools for assisting practitioners in classifying their patients’ femurs in daily practice.

Despite the alignment with the literature, our study has several limitations. These limitations include its retrospective design, relatively small sample size, suggesting limited power, as revealed by our post-hoc power analysis (material and method), and potential measurement errors.

Another crucial aspect and potential bias to consider in the use of the Amistem stem is its coating. Indeed, the coating has been modified in recent years to enhance osteointegration. The latest stem, which is proximally coated with plasma-sprayed titanium and hydroxyapatite, could improve the osteointegration of the stem, as discussed by Viamont-Guerra et al. in their recent article published in 2023 [19].

We also need to emphasize that our review focused solely on the radiological assessment of femoral stem evolution. It is important to note that the appearance of radiolucency does not always correlate with a failure of osteointegration or poor clinical outcomes. Engh et al. [10] described three types of fixation: stable fixation with bony ingrowth, unstable fixation, and stable fixation with fibrous ingrowth.

This latter type of fixation could be represented by patients with radiolucency and a good clinical outcome and cannot be screened out in our series. Finally, other limiting factors include the absence of information on the patient’s bone quality, which could have been provided by hip DEXA scans, as well as the short period of follow-up or survival data about the femoral stem.

Our results are not representative of other femoral stems used. A more comprehensive examination of the implant type used in Dorr A femurs is warranted, including the utilization of an anatomically designed stem with metaphyseal filling, which could enhance osteointegration in this configuration.

The strengths of this study lie in that all patients were operated by the same experienced surgeon using the same surgical technique, eliminating potential bias related to surgical experience and approach.

## Conclusions

Our study results are in consistency with recent research findings in terms of the osteointegration of femoral stems in Dorr type A femurs. The cortical index was the sole radiological parameter showing a significant correlation with an increased incidence of significant radiolucency in THA. Additionally, a threshold value was found to be associated with a greater incidence of early postoperative radiolucencies, with a cortical index ≥ 0.62. Although the sensitivity and specificity of this threshold are modest, it can still assist practitioners in planning elective THA for the choice of implants with respect to femoral morphology. However, it is crucial to note that further large-scale studies are imperative to the confirmation of these findings. A more comprehensive examination of the implant type used in Dorr A femurs is needed, including the utilization of an anatomically designed stem with metaphyseal filling, which could enhance osteointegration in this configuration.

## Data Availability

The data supporting the findings of this study are available upon request from the corresponding author (E.V.). Due to patient privacy, the data are not publicly accessible.

## Abbreviations

THA: Total hip arthroplasty
CI: Cortical index
CFI: Canal flair index
CC: Canal to calcar isthmus ratio
ICC: Intra-class correlation
BMI: Body mass index
